# Clinical characteristics of lactational breast abscess caused by methicillin-resistant *Staphylococcus aureus*: hospital-based study in China

**DOI:** 10.1186/s13006-021-00429-6

**Published:** 2021-10-12

**Authors:** Yan Li, Xiang-jun Ma, Xiang-ping He

**Affiliations:** Center for Prevention and Treatment of Breast Diseases, Haidian Maternal and Child Health Hospital, Beijing, China

**Keywords:** Breast abscess, Lactation, Methicillin-resistant *Staphylococcus aureus*, Needle aspiration, China

## Abstract

**Background:**

This study aimed to identify the differences in clinical characteristics, puncture efficacy, antibiotic use, treatment duration, breastfeeding post-illness, and recurrence of patients with breast abscesses caused by methicillin-resistant *Staphylococcus aureus* (MRSA) or methicillin-susceptible *Staphylococcus aureus* (MSSA) infection during lactation.

**Methods:**

The clinical data of patients with breast abscesses during lactation who were treated from January 2014 to February 2017 at Haidian Maternal and Child Health Hospital, Beijing, were reviewed. According to bacterial culture results, they were divided into MRSA (*n* = 260) and MSSA (*n* = 962) groups. Hospitalization (whether or not the patients were hospitalized), postpartum period, maternal age, location of abscess cavities, number of abscess cavities, amount of pus, frequency of needle aspiration, failure of needle aspiration, antibiotic use, treatment duration, cessation of breastfeeding and recurrence were compared between the two groups using a t-test and a chi-squared test.

**Results:**

We noted that only the cessation of breastfeeding was statistically significantly different between the two groups (*P* = 0.018). Hospitalization, postpartum period, maternal age, location of abscess cavities, number of abscess cavities, amount of pus, number of needle aspiration, failure of needle aspiration, antibiotic use, treatment duration and recurrence showed no statistically significant differences (*P* = 0.488, *P* = 0.328, *P* = 0.494, *P* = 0.218, *P* = 0.088, *P* = 0.102, *P* = 0.712, *P* = 0.336, *P* = 0.512, *P* = 0.386 and *P* = 0.359, respectively).

**Conclusions:**

There was no difference in clinical characteristics between breast abscesses infected by MRSA and those infected by MSSA. Ultrasound-guided needle aspiration could be the first choice for MRSA-infected breast abscess treatment. There is no need to increase antibiotic use because of MRSA infection, unless it is necessary. The reason why more patients with MRSA infected breast abscesses terminated breastfeeding is unclear from this study.

## Background

Breast abscesses are common in lactating women, with an incidence of 0.4 – 11% during lactation [1]. *Staphylococcus aureus* is the most common pathogenic bacteria among breast abscesses during lactation. It is a gram-positive bacteria with strong pathogenicity and can cause skin, soft-tissue, bone, joint, and systemic organ infections [2–5]. In 1961, the world’s first case of methicillin-resistant *Staphylococcus aureus* (MRSA) was isolated by Jevons [6]. The infection and isolation rates of MRSA have been increasing worldwide, and it has become one of the primary pathogens of nosocomial and community-acquired infections [7]. In recent years, the detection rate of MRSA in the breast milk and pus of lactating patients has gradually increased [2, 3, 5]. MRSA is characterized by high-level drug resistance and a complex drug resistance mechanism, which can increase infection-caused mortality, prolong the length of the hospital stay and increase medical expenses [8]. Therefore, many doctors might believe that patients with breast abscesses infected by MRSA are more serious and more difficult to treat. For the treatment, they may intervene more actively for MRSA than for a methicillin-susceptible *Staphylococcus aureus* (MSSA) infection. Is a MRSA-infected breast abscess a more serious condition than a MSSA-infected breast abscess? Does MRSA make it more difficult to treat breast abscesses? We conducted this study by collecting clinical data from 1222 patients to explore these issues.

## Methods

### Aim, design, and setting

Our team conducted preliminary analysis in 2019. Dr. Ding [9] retrospectively analyzed 174 patients in our hospital from January to July 2018, and found that there were no significant differences between MRSA and MSSA in abscess cavity location, abscess cavity size, abscess cavity number, antibiotic use and other factors. On the basis of the previous study, we expanded the sample size to 1525 patients in three years, and added more clinical factors for analysis, so as to explore whether there was any difference in the clinical manifestations of lactation breast abscess caused by these two bacteria.

We aimed to determine the clinical manifestations, aspiration efficacy, antibiotic use, treatment duration, breastfeeding and recurrence of patients with breast abscesses caused by MRSA or MSSA infection during lactation, using a large sample study, to guide the clinical diagnosis and treatment.

This was a retrospective study. We collected the clinical data on patients with breast abscesses during lactation treated in the Breast Disease Prevention and Treatment Centre of Haidian Maternal and Child Health Hospital, Beijing, from January 2014 to February 2017, using the electronic medical record system and reviewing medical records in the medical record room. All patients were followed up for one month by regular outpatient treatment and telephone follow-up.

### Patient characteristics

The inclusion criteria were as follows: (1) Patients diagnosed with breast abscesses during lactation who met all of the following criteria: lactating woman; breast lesions with inflammatory manifestations such as redness, swelling, heat and pain or accompanied by fever; physical examination showing palpable fluctuation; and a diagnostic breast ultrasound that would identify a collection of fluid. The collection would often be drained by needle aspiration, which would be diagnostic. (2) Microbiological results of aspiration of abscess were available. (3) The bacterial culture results showed MRSA or MSSA. Patients with no MRSA or MSSA or cases which there was no bacterial growth in the bacterial culture (*n* = 259) and patients lost to follow-up (*n* = 24) were excluded. (4) No other infections were present.

A total of 1525 patients with breast abscesses during lactation were treated in the Breast Disease Prevention and Treatment Centre of Haidian Maternal and Child Health Hospital, Beijing, from January 2014 to February 2017, of which 20 patients received no bacterial culture, 24 were lost to follow-up and 259 showed non-MRSA or non-MSSA in the bacterial culture. Thus, a total of 1222 patients met the inclusion criteria.

According to their bacterial culture results, all the patients were assigned to either the MRSA or MSSA group. Hospitalization, postpartum period, maternal age, location of abscess cavities, number of abscess cavities, amount of pus, number of needle aspiration, treatment method (failure of needle aspiration), antibiotic use, treatment duration, cessation of breastfeeding and recurrence were compared between the two groups. We aimed to determine whether the condition of patients with breast abscesses caused by MRSA infection was more serious than of those with MSSA infection and whether MRSA infection would prolong treatment duration or increase the recurrence rate. This study has been approved by the Ethics Committee of Beijing Haidian District Maternal and Child Health Hospital.

### Measures

Postpartum time referred to the time after the delivery of the patient. In our study the patients were divided into two groups as puerperium and non-puerperium, with postpartum 42 days as the cut-off point.

Location of abscess cavities referred to the position where the abscess appeared in the breast. In this study, the patients were divided into two groups: central area and non-central area. Central area meant the abscess appeared in the areola area of the breast.

Amount of pus was the maximum amount of pus aspirated in a single time during needle aspiration.

The cure criteria of needle aspiration were that the patients’ clinical symptoms disappeared, and the fluid aspirated by the last aspiration was non-purulent. The failure criteria of needle aspiration were skin ulcerations occurring after aspiration, the clinical symptoms not being relieved after aspiration and patients transferred to surgery.

The recurrence criteria were all patients followed up for one month without recurrence of clinical symptoms or another aspiration being considered clinically cured. Otherwise, the case was regarded as a recurrence.

Cessation of breastfeeding was the patient decided to terminate breastfeeding during the treatment period and did not resume breastfeeding after resolution of abscess.

### Treatment methods

**Fig. 1:**
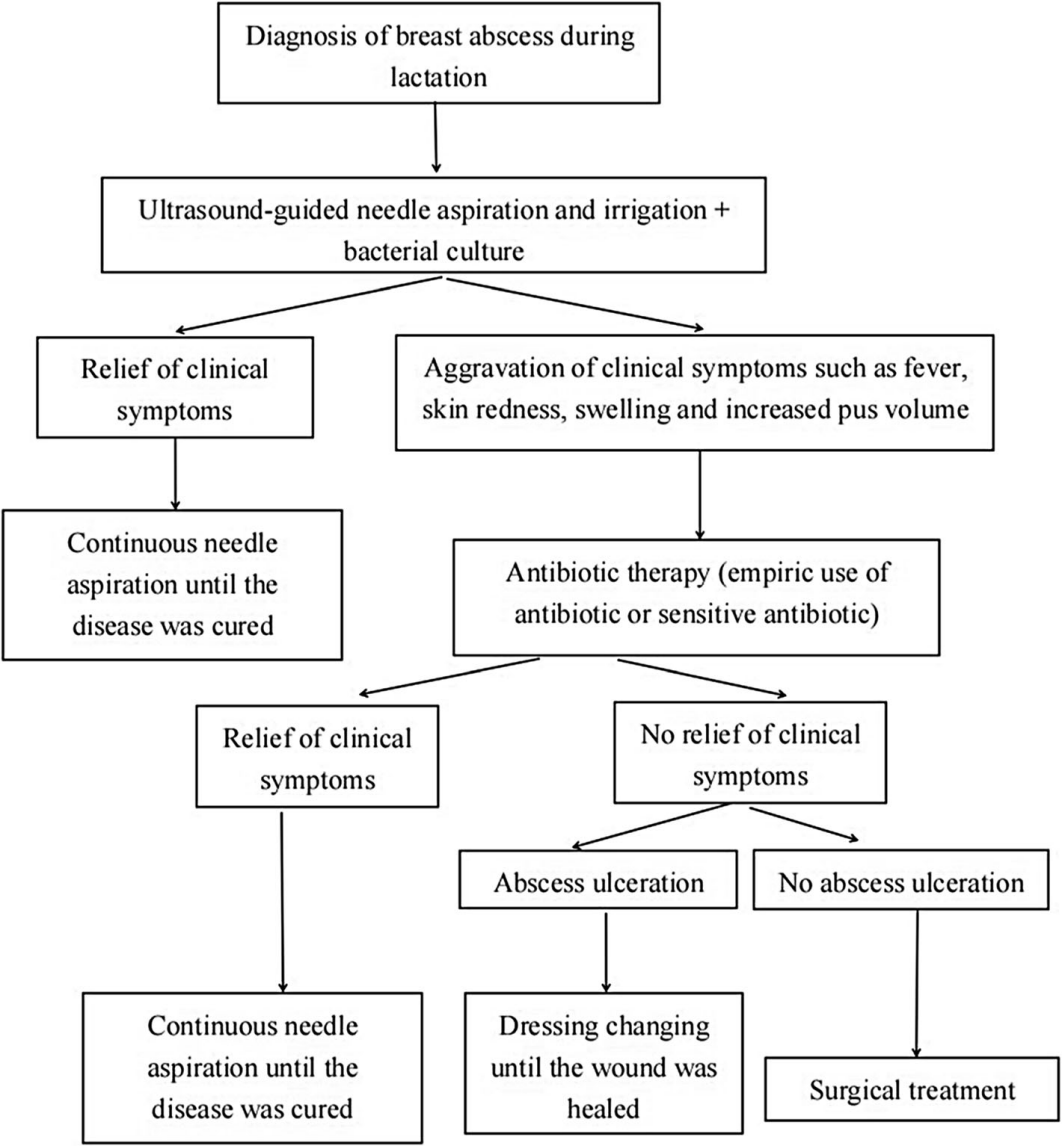
Flow chart of the treatment of breast abscess

All patients were treated with ultrasound-guided needle aspiration and irrigation for abscesses [10, 11]. If the clinical symptoms were not relieved after aspiration(such as continued fever, redness and swelling of the skin, and increased pus volume, adjuvant antibiotic therapy (empirical antibiotic or according to drug sensitivity results) would be given. If the clinical symptoms were still not relieved, surgical treatment was considered. Surgical methods included Mammotome minimally invasive vacuum-assisted biopsy of the abscess [12], abscess catheter irrigation and drainage or surgical incision and drainage (Fig. 1).

### Statistical analysis

Data were statistically analyzed using SPSS 21.0. Hospitalization, postpartum period, location of abscess cavities, number of abscess cavities, failure of needle aspiration, antibiotic use, weaning and recurrence were analyzed using the chi-squared test. Maternal age was analyzed by t-test. Amount of pus, frequency of needle aspiration and treatment duration were analyzed by Mann-Whitney U test.

## Results

From January 2014 to February 2017, 1525 patients were diagnosed with postpartum breast abscesses at our institution. Of the 1525 patients, 1481 (97.1%) had specimens sent for bacterial culture and 20 (1.3%) did not, 24 (1.6%) had no follow-up data. Of the 1481 patients’ specimens, 1284 isolated bacteria and 197 did not. The result is showed in Table 1. The 1222 patients with *Staphylococcus aureus* infection were the target people of our study, and the mean maternal age of them was 30.6 years old (21 ~ 44). Table 2 shows the result of Bacterial Sensitivities of MRSA.

**Table 1:** Distribution of organisms isolated from aspirates of 1481 patients with lactational breast abscess at Haidian Maternal and Child Health Hospital, Beijing from January 2014 to February 2017

Organism isolated^**a**^	Number	Detection rate
No pathogenic bacteria	197	13.3%
*Staphylococcus aureus*	1222	82.5%
MSSA^b^	962	65.0%
MRSA^c^	260	17.6%
*Staphylococcus epidermidis*	20	1.4%
*Streptococcus mitis*	8	0.5%
*Streptococcus sanguinis*	5	0.3%
*Staphylococcus intermedia*	4	0.3%
*Streptococcus salivarius*	4	0.3%
*Streptococcus pyogenes*	3	0.2%
*a-hemolytic streptococcus*	2	0.1%
*Streptococcus agalactiae*	2	0.1%
*Staphylococcus haemolyticus*	2	0.1%
*Candida albicans*	1	0.07%
*Escherichia coli*	1	0.07%
*Klebsiella pneumoniae*	1	0.07%
*Streptococcus pneumoniae*	1	0.07%
*Enterococcus faecalis*	1	0.07%
*Streptococcus pharyngitis*	1	0.07%
*Staphylococcus cephalus*	1	0.07%
*Pseudomonas aeruginosa*	1	0.07%
*Staphylococcus sciuri*	1	0.07%
*Lactococcus Lactis*	1	0.07%
*Acinetobacter lwoffii*	1	0.07%

^a^. Some aspirates from patients had more than one organism isolated, but only the pathogenic bacteria are reported (*Staphylococcus epidermidis* only reported if no other organism isolated)

^b^. *MSSA* methicillin-susceptible *Staphylococcus aureus*

^c^. *MRSA* methicillin-resistant *Staphylococcus aureus*

**Table 2:** Bacterial sensitivities of 260 MRSA isolates from lactational breast Abscesses at Haidian Maternal and Child Health Hospital, Beijing, from January at Haidian Maternal and Child Health Hospital, Beijing, from January 2014 to February 2017

Antibiotic	Sensitive ***n*** (%)	Resistant ***n*** (%)
Vancomycin	260 (100%)	0 (0%)
Nitrofurantion	253 (97.3%)	7 (2.7%)
Levofloxacin	250 (96.2%)	10 (3.8%)
Gentamicin	250 (96.2%)	10 (3.8%)
Trimethoprim-sulfamethoxazole	240 (92.3%)	20 (7.7%)
Tetracycline	143 (55%)	117 (45%)
Clindamycin	39 (15%)	221 (85%)
Erythromycin	34 (13.1%)	226 (82.9%)
Cefoxitin	0 (0%)	260 (100%)
Amoxicillin-clavulanate	0 (0%)	260 (100%)
Penicillin	0 (0%)	260 (100%)

*MRSA* methicillin resistant *Staphylococcus aureus*

A total of 91 patients (7.4%) terminated breastfeeding after resolution of the abscess. The cessation of breastfeeding rate of the MRSA group was 10.8% (28/260), and that of the MSSA group was 6.5% (63/962). Comparisons using chi-square test showed a *p* - value of 0.018, with significant statistical difference between the two groups.

Among the 1222 patients, the average number of repeated aspirations were 2.9 per patient and the average volume of pus was 17.6 ml. And there was no statistical difference in frequency of needle aspiration and the amount of pus between the two groups (Table 3). There were 1132 patients (92.6%) who were cured by repeated ultrasound-guided needle aspiration. Another 90 patients failed to respond after aspiration treatment, including 20 patients who underwent catheter irrigation and drainage, 15 patients underwent Mammotome minimally invasive vacuum-assisted biopsy, 10 patients underwent surgical incision and drainage and 20 patients who developed abscess ulceration recovered by cleaning the wound. Of these 90 patients, 17 (6.5%, 17/260) were in the MRSA group and 73 (7.6%, 73/962) were in the MSSA group. There was no statistical difference in the failure of needle aspiration between MRSA and MSSA groups (Table 4).

**Table 3:** Comparison of age, amount of pus, number of aspiration and treatment duration between the MRSA group and MSSA group

Variable	MRSA group (***n*** = 260)		MSSA group (***n*** = 962)		***P*** - value
	Median	Mean	Median	Mean	
Maternal age, year (range)	30 (21–44)	30.6	30 (20–48)	30.6	0.494^a^
Amount of pus, mL (range)	8 (0–143)	16.5	7 (0–420)	17.9	0.556^b^
Number of aspirations, times (range)	3 (0–12)	3.0	3 (0–16)	2.9	0.126^b^
Treatment duration, days (range)	4 (1–90)	7.9	4 (1–85)	7.8	0.441^b^

^a^. T-test

^b^. Mann-Whitney U test

**Table 4:** Comparison of the characteristics of participants in which MRSA and MSSA were isolated from their lactational breast abscess

Variables	MRSA group (***n*** = 260)	MSSA group (***n*** = 962)	χ^**2**^	***P*** - value
Hospitalization (%)			0.012	0.488
Residential treatment	44 (16.9%)	160 (16.6%)		
Outpatient treatment	216 (83.1%)	802 (83.4%)		
Postpartum period (%)			0.269	0.328
Puerperium^a^	149 (57.3%)	534 (55.5%)		
Non-puerperium	111 (42.7%)	428 (44.5%)		
Location of abscess cavities (%)			0.738	0.218
Central area^b^	66 (25.4%)	270 (28.1%)		
Non-central area	194 (74.6%)	692 (71.9%)		
Number of abscess cavities (%)			2.063	0.088
Single cavity	180 (69.2%)	709 (73.7%)		
Multiple cavities^c^	80 (30.8%)	253 (26.3%)		
Failure of aspiration^d^ (%)			0.331	0.336
Failed	17 (6.5%)	73 (7.6%)		
Non-failed	243 (93.5%)	889 (92.4%)		
Antibiotic use (%)			0.003	0.512
Yes	82 (31.5%)	305 (31.7%)		
No	178 (68.5%)	657 (68.3%)		
Cessation of breastfeeding (%)			5.290	0.018
Cessation	28 (10.8%)	63 (6.5%)		
Non-cessation	232 (89.2%)	899 (93.5%)		
Recurrence^e^(%)			0.390	0.359
Recurrent	5 (1.9%)	25 (2.6%)		
Non-recurrent	255 (98.1%)	937 (97.4%)		

^a^Puerperium: within 42 days of giving birth.

^b^Central area: the abscess located in the areola area.

^c^Multiple cavities: the number of cavities are more than 1.

^d^Failure of aspiration: the patient’s symptoms do not resolve and are eventually treated with other surgical methods.

^e^Recurrence refers to the recurrence of an abscess at the original site within one month

There were 387 patients treated with antibiotics. Of these, 243 patients received cephalosporins, 108 patients received levofloxacin, 14 patients received gentamycin, 7 patients received clindamycin, 6 patients received etimicin, 4 patients received azithromycin, 2 patients received penicillin, 2 patients received vancomycin, and 1 patient received ertapenem. Eighty-seven patients in the MRSA group received antibiotic treatment (31.5%, 82/260), and 305 patients received antibiotics in the MSSA group (31.7%, 305/962). Of the 82 patients in the MRSA group who received antibiotics, 37 patients did not receive sensitive antibiotics due to delayed bacterial culture results. Thirty-four patients recovered after being treated by ultrasound-guided needle aspiration. For the remaining patients, 1 underwent catheter irrigation and drainage, 1 was cured after Mammotome minimally invasive vacuum-assisted biopsy and 1 recovered by cleaning the wound who appeared abscess ulceration. Among the 178 (68.5%, 178/260) patients with non-antibiotic treatment in MRSA infection group, 174 patients were cured by ultrasound-guided needle aspiration, and the 4 people failed to respond; 2 patients appeared ulcerated, one person underwent Mammotome minimally invasive vacuum-assisted biopsy and one person treated by catheter irrigation and drainage.

In this study, 683 of 1222 patients were puerperal which was within 42 postpartum days: 149 were in the MRSA group and 534 were in the MSSA group (no difference between groups; Table 4). We also compared other abscess features between the two groups, such as maternal age, hospitalization rate, abscess cavity location, the number of cavities, treatment duration and recurrence rate. This study found no evidence of differences in the above features between MRSA group and MSSA group (Table 3, Table 4).

## Discussion

In recent years, the detection rate of MRSA in lactating patients has gradually increased [2, 3, 5]. Whether the patients with MRSA-infected breast abscesses are more serious than those infected with MSSA or whether MRSA increases the difficulty of breast abscess treatment remains unclear. Presently, there are few studies on these aspects, and the sample sizes are small. So, we conducted this research to explore those questions.

Our study showed that only the cessation of breastfeeding was significantly different between patients with breast abscesses during lactation in MRSA and MSSA groups. The cessation of breastfeeding rate in MRSA group was higher than that in MSSA group (10.7% vs 6.5%), which indicated that patients with MRSA-infected breast abscesses during lactation were more likely to terminate breastfeeding. All patients in our study were advised to continue breastfeeding during treatment. Even if breastfeeding was stopped while taking the medications, breastfeeding could continue after the drugs were stopped. However, Reddy’s study [13] showed a different result. Their study showed that the cessation of breastfeeding rate after mastitis in the MRSA group was 16% and that in the MSSA group was 22%, without a statistical significance. The authors think this may be related to the use of antibiotics which are perceived as unsafe during breastfeeding in MRSA group. Most of the patients in the MRSA group were given levofloxacin and vancomycin, and breastfeeding was temporarily stopped during the treatment in China, while most of the patients in the MSSA group were given cephalosporins and continued breastfeeding. After the temporary interruption of breastfeeding, some mothers are not willing to return to breastfeeding. So, we thought interruption of breastfeeding by using antibiotics might increase the cessation of breastfeeding rate in MRSA group. Moreover, some patients worried that MRSA infection would prolong the recovery time of the disease, and wanted to avoid the recurrence of abscesses through terminating breastfeeding. This may be another reason for the high cessation of breastfeeding rate in the MRSA group. Our results showed that patients with MRSA-infected breast abscesses were more likely to terminate breastfeeding, and it was a patients’ decision. This does not mean that MRSA affects breastfeeding. A study in Taiwan [14] reported a colonization rate of 8.1% MRSA in the nasal cavity of healthy children. They analyzed the risk factors for MRSA and MSSA carriage, and found that breastfeeding, *S. pneumoniae* colonization, and upper respiratory tract infection within two weeks were protective factors against MSSA colonization, while only breastfeeding was a protective factor against MRSA colonization. Therefore, breastfeeding can still be continued in patients with MRSA-infected breast abscesses during lactation [13, 15]. We can see the breastfeeding rate of the two groups in our study were both higher than that of Reddy’s study [13] and it shows the efforts of Chinese doctors to advance breastfeeding in recent years. However, now in China, the rate of breastfeeding is still below the world average and we have a lot of work to do to improve the breastfeeding rate in China.

With the development of minimally invasive treatment technology, ultrasound-guided needle aspiration has become the preferred treatment method for breast abscesses and is widely used in clinical practice [11, 16, 17]. In our study, repeated ultrasound-guided needle aspiration resolved the problem in 1132 of 1222 participants, with a cure rate of 92.6%. The failure of ultrasound-guided needle aspiration was not significantly different between MRSA and MSSA groups, so MRSA infection did not increase the failure rate of ultrasound-guided needle aspiration. This result is consistent with the study by Chen CY et al. [18]. Overall, our study supports the use of ultrasound-guided needle aspiration as the first choice for MRSA-infected breast abscess treatment.

Lam et al. [19] reviewed the literature on the treatment of breast abscesses, and recommended that all patients with breast abscesses should be treated concurrently with antibiotics. However, according to Luo and colleagues [20], antibiotics were not routinely used for the treatment of breast abscesses during lactation, but the success rate was similar to that of routine antibiotic use. Our research showed a 31.7% antibiotic utilization rate. The statistical analysis demonstrated no difference between the MRSA and MSSA groups (31.5%, 82/260 vs 31.7%, 305/962), suggesting that MRSA infection in breast abscesses during lactation did not increase the use of antibiotics. Considering the above results, the use of antibiotics is not the first choice for the treatment of breast abscess, and there is no need to increase the use of antibiotics because of MRSA infection. Therefore, for the treatment of breast abscesses during lactation, effective drainage of pus might be necessary. Without drainage of infected fluid, the use of antibiotics is ineffective [21]. There is little consensus on when to begin antibiotics. Our treatment experience is that antibiotic treatment is not necessary when patient’s symptoms are significantly relieved after aspiration or drainage. Patients with more severe systemic inflammation or poor result after aspiration or drainage treatment should be treated with antibiotics. In China, if MRSA-sensitive antibiotics such as levofloxacin or vancomycin are used, patients are advised to stop breastfeeding as the Chinese package inserts of the two drugs state that breastfeeding should be stopped during lactation. Reducing the use of antibiotics is a medical support for breastfeeding which can help improve breastfeeding rates in China. Now antibiotic resistance is a global problem. Antibiotic overuse triggers the spread of resistant strains in the population. As a result, many strains of dangerous bacteria pathogens nowadays are resistant to antibiotics, with some strains combining even multiple resistances to different antibiotics. Possibly the most infamous example of an antibiotic-resistant pathogen is *Staphylococcus aureus*. Therefore, reducing the use of antibiotics can also help reduce the emergence of antibiotic-resistant strains [22, 23].

Meanwhile, we found that 37 patients in the MRSA group did not receive sensitive antibiotics due to delayed bacterial culture results and they were all cured. The result puzzled us about the best time to use antibiotics. For these 37 patients, if we had more frequent needle aspiration therapy or earlier surgery, would we be able to cure them without antibiotics? By now our research can’t answer the question, but the result is worth further investigation. And it also implies that it is not necessary to change the sensitive antibiotics when the patient’s symptoms are improving, even if the insensitive antibiotics are used.

Our study shows that there were no statistically significant differences in the duration of treatment and the infection recurrence rate, which is consistent with the results of Chen CY et al. [18]. Our study also showed that MRSA infection did not increase the recurrence rate or prolong the treatment duration of patients with lactational breast abscesses compared with MSSA infection.

In this study, no significant differences were found in age, postpartum period, hospitalization rate, abscess cavity location, the number of abscess cavities and the amount of pus between patients with breast abscesses caused by MRSA infection or MSSA infection during lactation. The result was same with other studies [9, 13, 18].

Overall, in our study, patients who were infected by MRSA did not experience poorer treatment outcomes, as measured by duration of treatment, rate of recurrence, compared with those infected by MSSA. And there was no difference in clinical characteristics between breast abscesses infected by MRSA and those infected by MSSA, as assessed by maternal age, postpartum period, hospitalization rate, abscess cavity location, the number of abscess cavities and the amount of pus.

Although the sample size in this study was large, it was a retrospective study and has some limitations. The lack of criteria for antibiotic use, standard conversion from needle aspiration to surgery, and patient’s willingness to wean may affect the conclusions of the study. We also used the disc diffusion method to test MRSA in our study and this method can’t distinguish community-associated MRSA and healthcare-associated MRSA strains, so it is a limitation of the study. Therefore, the results should be further confirmed by large sample prospective studies.

## Conclusions

This study shows that patients with MRSA-infected breast abscesses during lactation were more likely to terminate breastfeeding. Our study also supports the use of ultrasound-guided needle aspiration as the first choice for MRSA-infected breast abscess treatment and the use of antibiotics is not the first choice for the treatment of breast abscess infected by MRSA. There is no need to increase antibiotic use because of MRSA infection unless it is necessary. Moreover, there was no difference in clinical characteristics between breast abscesses infected by MRSA and those infected by MSSA.

## Data Availability

The data sets used and/or analyzed during the current study are available from the corresponding author on reasonable request.

## Abbreviations

MRSA: Methicillin-resistant *Staphylococcus aureus*
MSSA: Methicillin-susceptible *Staphylococcus aureus*
